# Early Versus Late Initiation of Hydrocortisone in Patients With Septic Shock: A Prospective Study

**DOI:** 10.7759/cureus.50814

**Published:** 2023-12-19

**Authors:** Moath Alsulami, Lamees Alrojaie, Abubker Omaer

**Affiliations:** 1 Pharmacy, Umm Al-Qura University, Makkah, SAU; 2 Pharmacy, King Saud Medical City, Riyadh, SAU

**Keywords:** survival, ventilator, late, early, mortality, hydrocortisone, icu, corticosteroids, septic shock, sepsis

## Abstract

Introduction

The optimal timing of corticosteroid initiation in septic shock patients is debatable. The Surviving Sepsis Campaign Guidelines recommended adding hydrocortisone to septic shock patients who require a vasopressor with a dose of norepinephrine ≥ 0.25 mcg/kg/min for at least four hours. Nevertheless, the best time to initiate hydrocortisone remains uncertain.

Objective

Assessing the impact of early (≤3 hours) versus late (>3 hours) initiation of hydrocortisone in septic patients.

Methodology

We compared the outcomes of septic shock patients who received hydrocortisone within three hours versus those who started treatment after three hours. The inclusion criteria encompassed septic shock patients aged 18 or older who received at least one dose of hydrocortisone. Exclusion criteria included pregnancy, do-not-resuscitate orders, the absence of empirical intravenous antibiotics, recent corticosteroid use, recent cardiac arrest, and a history of adrenal insufficiency.

Results

Eighty-one patients were included (54% were males). The mean age was 59 years, and 56.8% of patients were in the early group. The time needed to discontinue vasopressors was 25 and 37 hours for the early and late groups, respectively (p = 0.009), and more patients achieved reversal of shock (35 vs. 24 patients) and had shorter ICU stays (17 days vs. 20 days).

Conclusion

Initiating hydrocortisone early, within three hours, reduced the time needed to discontinue vasopressors among the study population. However, both early and late initiation strategies yielded comparable outcomes in terms of ICU mortality, ICU length of stay, and shock reversal.

## Introduction

Septic shock is clinically defined as a manifestation of sepsis, presenting with persistent hypotension that necessitates the use of vasopressors to maintain a mean arterial pressure (MAP) of 65 mm Hg, along with serum lactate levels exceeding 18 mg/dL (2 mmol/L) despite appropriate volume resuscitation [1]. Despite treatment progress, septic shock continues to pose a significant global challenge, affecting millions of individuals annually. In the United States, the estimated percentage mortality rate among critically ill patients with septic shock exceeds 40% [2-4].

In managing shock and restoring hemodynamic stability, intravenous (IV) fluid resuscitation and vasoactive agents are commonly employed [3]. However, prolonged use of IV vasopressors has been associated with increased length of stay (LOS) and higher mortality rates [4]. In cases where fluid resuscitation and IV vasopressor therapy are insufficient for achieving hemodynamic stability, both the Surviving Sepsis Campaign (SSC) and the American College of Critical Care Medicine recommend the administration of hydrocortisone in doses up to 200 mg/day. Nevertheless, the strength of these recommendations remains a subject of debate due to uncertainties surrounding the efficacy of hydrocortisone in treating septic shock [3].

Corticosteroids play a role in modulating the dysregulated host response by decreasing the expression of proinflammatory mediators, receptors, and cytokines [5,6]. Among corticosteroids, hydrocortisone is commonly used in the treatment of septic shock due to its glucocorticoid and mineralocorticoid activity [5,6]. Mineralocorticoids primarily affect ion transport in renal tubule epithelial cells and are involved in fluid and electrolyte management through the renin-angiotensin-aldosterone pathway. Activation of this pathway stimulates aldosterone secretion, resulting in increased salt and water retention and elevated blood pressure. Glucocorticoids, on the other hand, mainly exhibit anti-inflammatory and vasoconstrictive properties. As a result, low-dose hydrocortisone has been proposed as an adjunctive therapy to vasopressors in septic shock patients. Furthermore, hydrocortisone improves hemodynamic parameters by increasing vascular sensitivity and reducing vasopressor requirements [7].

Several studies have reported a correlation between the timing of corticosteroid therapy initiation in septic patients and key outcomes such as the time of vasopressor discontinuation, shock reversal, intensive care unit (ICU) length of stay, and mortality. However, the optimal timing for initiating such an intervention remains a matter of debate. The aim of this study is to compare the outcomes of septic shock patients who received hydrocortisone within three hours (early group) with those who started treatment later, beyond three hours (late group).

## Materials and methods

This prospective observational cohort study was conducted at King Saud Medical City, a tertiary hospital in Riyadh, Saudi Arabia, between February and August 2022. Patients were divided into two groups based on the timing of hydrocortisone initiation from diagnosis: the early group (within three hours) and the late group (after three hours). The primary outcome assessed was the time required for vasopressor discontinuation, while the secondary outcomes included shock reversal (patients are hemodynamically stable without vasopressors for 24 hours), ICU mortality, and ICU length of stay. The study included all septic shock patients aged 18 or older who received at least one dose of hydrocortisone. Exclusion criteria comprised patients below 18 years old, pregnant women, patients with do-not-resuscitate (DNR) orders, individuals not receiving empirical IV antibiotics, those who had received corticosteroids or experienced cardiac arrest within the previous 30 days, and patients with a history of adrenal insufficiency.

Institutional review board (IRB) approval

The study has been granted IRB approval from King Saud Medical City. The IRB approval was under the code H01R053.

Statistical analysis

The primary outcome was analyzed using Kaplan-Meier curves and the Wilcoxon log-rank test. The normality of numerical variables was assessed using the Shapiro-Wilk test, and the Mann-Whitney U test and T-test were employed to compare continuous variables according to normality. The association between the late and early administration of hydrocortisone and categorical variables was examined using chi-square and Fisher's exact tests. All the statistical analyses were conducted using StataCorp. 2021. Stata Statistical Software: Release 17. College Station, TX: StataCorp LLC.

## Results

The normality of the numerical variables was assessed using the Shapiro-Wilk test, revealing that all variables, except age, height, weight, MAP, and BMI, were found to be non-normally distributed (p < 0.05). Table 1 presents the baseline characteristics, demonstrating a relatively equal representation of males and females in the study. The patients had a mean age of 59 years, with median SOFA score of 3, a mean MAP of 65 mmHg, and an initial lactic acid level of 2.6 mmol/L, originating from various sources of infection.

**Table 1:** Baseline characteristics

	Total	Late	Early	p-value
	N=81	N=35	N=46	
Age, (mean, SD)	60 (18)	56 (18)	63 (17)	0.077
Gender (n, %)	44 (54%)	21 (60%)	23 (50%)	0.37
Weight (Kg), (mean, SD)	77 (20)	78 (25)	76 (16)	0.66
Height, (mean, SD)	164 (10)	165 (9)	164 (10)	0.51
Body mass index (mean, SD)	28 (7)	28 (8)	28 (6)	0.8
SOFA Score (mean, SD)	3 (3-3)	3 (3-3)	3 (2-3)	0.7
Initial Lactic acid (mmol/L) (mean, SD)	3 (2-4)	3 (2-4)	3 (2-3)	0.09
Mean arterial pressure (mean, SD)	65 (5)	65 (6)	65 (5)	0.78
Hypertension (n, %)	51 (63%)	20 (57%)	31 (69%)	0.28
Diabetes (n, %)	48 (59%)	20 (57%)	28 (62%)	0.65
Chronic obstructive pulmonary disease (n, %)	2 (0.02%)	1 (0.03%)	1 (0.02%)	0.86
Heart failure (n, %)	4 (5%)	2 (6%)	2 (4%)	0.8
COVID (n, %)	3 (4%)	1 (3%)	2 (4%)	0.71
Need for mechanical ventilation (n, %)	77 (96%)	34 (97%)	43 (96%)	0.71
Need for renal replacement (n, %)	24 (30%)	11 (31%)	13 (28%)	0.76
Reversal of shock (n, %)	59 (73%)	24 (69%)	35 (76%)	0.45
Respiratory source of infection (n, %)	50 (62%)	21 (60%)	29 (63%)	0.78
Intra-abdominal source of infection (n, %)	8 (10%)	5 (14%)	3 (7%)	0.25
Urinary source of infection (n, %)	21 (26%)	10 (29%)	11 (24%)	0.64
Skin-soft tissue source of infection (n, %)	14 (17%)	6 (17%)	8 (17%)	0.98
Bacteremia source of infection (n, %)	31 (38%)	12 (34%)	19 (41%)	0.52
Other sources of infection (n, %)	1 (3%)	1 (5%)	0 (0%)	0.32
ICU-mortality (n, %)	44 (54%)	19 (54%)	25 (54%)	1

Among the patients, 56.8% received early hydrocortisone treatment, while 43.2% received it later. The median duration of vasopressor usage was 113 hours, with a median of one vasopressor required. The median length of stay (LOS) in the ICU was 23 days; in the hospital, it was 44 days. Additionally, the patients had a median duration of mechanical ventilation of 17.5 days.

**Figure 1:**
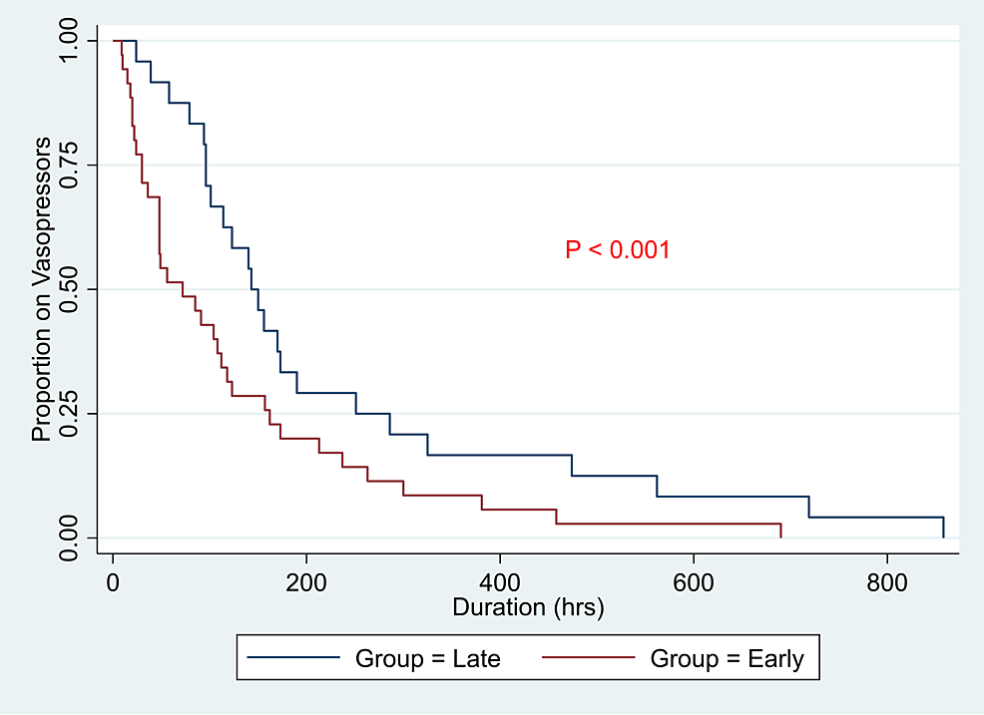
Time to discontinue vasopressors

In Figure 1, the time to discontinuation of vasopressors is presented. The median time to discontinue vasopressors is significantly different between the early and late groups (72 hours and 143 hours, p<0.001).

Table 2 presents the results of the Mann-Whitney U test, which aimed to compare the time of vasopressor use between the early and late corticosteroid administration groups. The analysis revealed a statistically significant difference between the groups (U = 250, p = 0.009), with a higher mean rank observed in the late group (37) compared to the early group (25). However, when assessing the outcome of "day alive and ventilator-free at 28 days," no statistically significant difference was found between the groups (U = 32.5, p = 0.116). The mean rank was lower in the late group (17) compared to the early group (11.7). Additionally, no statistically significant differences were observed between the late and early groups regarding the following outcomes: days alive and vasopressor-free at 28 days, ICU length of stay, hospital length of stay, number of needed vasopressors, and number of days on a ventilator.

**Table 2:** A comparison between the primary and secondary outcomes of the early and late groups was conducted using the Mann-Whitney U test

Outcome variable	Groups	Mean rank	U value	P-value
Number of hours on vasopressor	Late	37	250	0.009
	Early	25		
Days alive & free from vasopressor at 28-day	Late	39	718	0.362
	Early	43		
Days alive & free from the ventilator at 28-day	Late	17	32.5	0.116
	Early	11.7		
ICU length of stay (days)	Late	20	131	0.355
	Early	17		
Hospital length of stay (days)	Late	21	111	0.193
	Early	16		
Number of vasopressors needed	Late	41 (median=1, IQR=1)	795	0.911
	Early	41 (median=1, IQR=1)		
Number of days on mechanical ventilation	Late	23	122	0.109
	Early	17		

Statistical tests, including chi-square and Fisher's exact test, were employed to examine the association between the late and early administration of hydrocortisone and categorical variables. The analysis revealed no statistically significant associations between the timing of corticosteroid administration (late or early) and variables such as shock reversal, need for renal replacement therapy, or ICU mortality. Table 3 presents the detailed results of these analyses.

**Table 3:** Association between timing of corticosteroid administration and secondary outcomes

		Corticosteroid administration		
		Late	Early	p-value
Need for renal replacement therapy (n, %)	No	24 (68.6%)	33 (71.7%)	0.757
	Yes	11 (31.4%)	13 (28.3%)	
shock reversal (n, %)	No	11 (31.4%)	11 (23.9%)	0.463
	Yes	24 (68.6%)	35 (76.1%)	
ICU mortality (n, %)	No	16 (45.7%)	21 (45.7%)	1
	Yes	19 (54.3%)	25 (54.3%)	

## Discussion

The optimal timing for administrating hydrocortisone to patients with septic shock remains a subject of ongoing debate. In a randomized controlled trial conducted by Annane et al. in 2002 [8], low-dose corticosteroids were investigated for their effect on 28-day survival in patients with septic shock and adrenal insufficiency. The patients were randomized within eight hours of septic shock onset, and those with adrenal insufficiency showed improved survival and shock reversal following treatment with hydrocortisone and fludrocortisone (P = 0.001). In contrast, the CORTICUS trial examined the safety and efficacy of low-dose hydrocortisone administered within 72 hours of septic shock onset. Although hydrocortisone did not improve 28-day survival, it did accelerate shock reversal (P = 0.69) [6].

Furthermore, in a recent randomized controlled trial, Lv et al. found no significant differences in shock reversal or mortality when hydrocortisone was initiated concurrently with IV vasopressors [9]. In a retrospective study published in 2012, Park et al. demonstrated that low doses of hydrocortisone administered within six hours of initiating vasopressors significantly reduced mortality compared to administration after six hours (P = 0.0132) [4]. Katsenos et al. conducted a prospective study in which the time to vasopressor withdrawal was significantly shorter in patients receiving hydrocortisone within nine hours than those receiving it after 10 hours (P < 0.001) [5]. The different cut-off points used among the studies and our study might lead to differences in mortality results and shock resolution. However, the exact impact and timing need to be investigated in large direct comparison studies adjusted for underlying comorbidities.

Additionally, the early administration group exhibited a statistically significant mortality advantage (P = 0.029), indicating that the timing of hydrocortisone initiation impacts survival in septic shock patients [5]. Furthermore, in a retrospective study published in 2021, Ragoonanan D et al. found that administering hydrocortisone within 12 hours of septic shock onset was associated with a faster time to vasopressor withdrawal, as well as shorter ICU and hospital stays (P = 0.0132) [10].

In our study, we aimed to investigate the impact of the timing of hydrocortisone administration (early or late) on the clinical outcomes of critically ill patients. Our findings revealed that patients who received early intravenous hydrocortisone (within three hours) had a significantly shorter time to discontinue vasopressors than those who received late intravenous corticosteroids. However, we did not observe significant differences in ICU mortality, shock reversal, ventilator-free days, ICU length of stay, hospital length of stay, or the number of required vasopressors. The observed effect on the time to vasopressor discontinuation aligns with the findings of previous studies [5,10] by Ragoonanan et al. and Katsenos et al. However, it is worth noting that the definition of early intravenous corticosteroid administration varied across these studies. Our study considered it within three hours, while Ragoonanan et al. defined it as less than 12 hours, and Katsenos et al. used a threshold of nine hours [5,10].

We did not find significant differences in the other clinical outcomes, contrasting with the results reported by Ragoonanan et al., who observed shorter ICU and hospital lengths of stay in the early intravenous corticosteroid group [10]. The disparity in these findings could be attributed to the smaller sample size in our study.

Regarding mortality, our study and the study by Ragoonanan et al. did not identify a difference between the early and late administration groups [10]. However, Katsenos et al. reported decreased mortality in the early group [5]. These discrepancies may be attributed to variations in baseline characteristics among the patient populations across the studies. Consistent with the results [10] of Ragoonanan et al., we did not find a significant difference in the number of required vasopressors or the need for renal replacement therapy between the early and late administration groups. In terms of shock reversal, our study did not reveal a significant difference between the groups, while Ragoonanan et al. reported a significantly improved shock reversal with earlier initiation of hydrocortisone [10].

The agreement and disagreement among the studies regarding the comparison of early and late intravenous corticosteroid initiation underscore the importance of establishing a unified definition for shock, shock reversal, and the optimal timing of "early" initiation. This standardization would guide clinical practice. Additionally, the heterogeneity of the patient populations may have influenced the results and contributed to discrepancies in the outcomes.

Our study has certain limitations. First, the sample size is relatively small; however, our results align with findings from larger studies. Second, we did not employ propensity score matching; nevertheless, the baseline characteristics table demonstrated no significant differences between the study groups, except for age. Larger studies are needed in this area, especially to highlight the optimal timing of corticosteroid initiation in shock patients, considering the heterogeneity in this critical population.

## Conclusions

In conclusion, early initiation of hydrocortisone (within three hours) appears to have favorable effects on clinical outcomes in critically ill patients, particularly in terms of timing for discontinuing vasopressors. However, additional, larger studies are needed to provide more comprehensive guidance for clinical practice.
